# Supplementary material for: Low-Dose Administration of Cannabigerol Attenuates Inflammation and Fibrosis Associated with Methionine/Choline Deficient Diet-Induced NASH Model via Modulation of Cannabinoid Receptor
Source: Nutrients. 2022 Dec 30;15(1):178. doi: 10.3390/nu15010178 (PMC9823433; doi:10.3390/nu15010178)
Supplement: Supplementary file 1 [file nutrients-15-00178-s001.zip › nutrients-2041396-supplementary.pdf]

Supplemental Figure S1

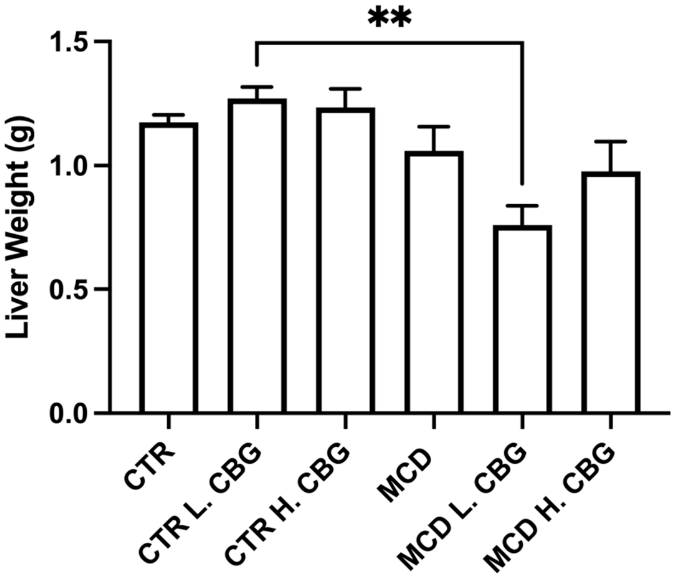

Supp Figure S1. Evaluating the overall health of mice or without NASH diet induction. The Liver weight was assessed after sacrifice. **p<0.01

Supplemental Figure S2

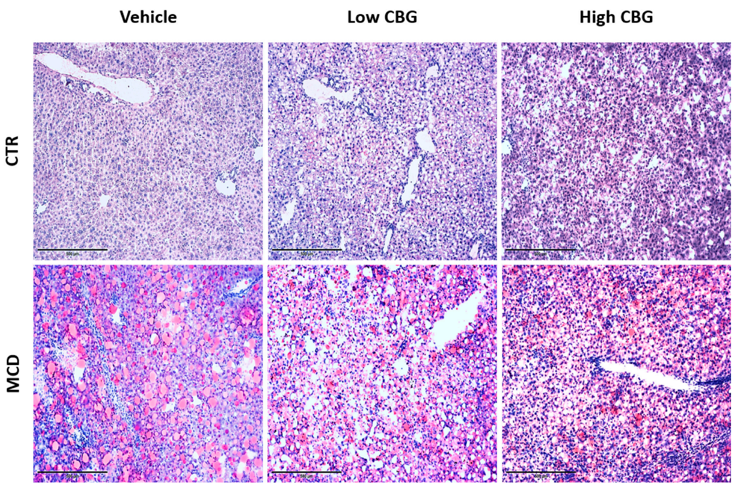

Supp figure S2. Evaluating steatosis. Representative images of Oil-Red-O staining (20X) illustrating fat deposition in frozen liver sections.
